# Systemic immunological responses are dependent on sex and ovarian hormone presence following acute inhaled woodsmoke exposure

**DOI:** 10.1186/s12989-024-00587-5

**Published:** 2024-05-27

**Authors:** Kartika Wardhani, Sydnee Yazzie, Charlotte McVeigh, Onamma Edeh, Martha Grimes, Quiteria Jacquez, Connor Dixson, Edward Barr, Rui Liu, Alicia M. Bolt, Changjian Feng, Katherine E. Zychowski

**Affiliations:** 1 College of Nursing, University of New Mexico-Health Sciences Center, Albuquerque, New Mexico USA; 2 Biochemistry and Biotechnology Group (B-TEK), Bioscience Division, Los Alamos National Laboratory, Los Alamos, New Mexico USA; 3 Department of Pharmaceutical Sciences, College of Pharmacy, University of New Mexico-Health Sciences Center, Albuquerque, New Mexico USA

**Keywords:** Wildfire, Woodsmoke, Sex-differences, Ovariectomized, Immune, Cancer

## Abstract

**Background:**

Rural regions of the western United States have experienced a noticeable surge in both the frequency and severity of acute wildfire events, which brings significant challenges to both public safety and environmental conservation efforts, with impacts felt globally. Identifying factors contributing to immune dysfunction, including endocrinological phenotypes, is essential to understanding how hormones may influence toxicological susceptibility.

**Methods:**

This exploratory study utilized male and female C57BL/6 mice as in vivo models to investigate distinct responses to acute woodsmoke (WS) exposure with a focus on sex-based differences. In a second set of investigations, two groups were established within the female mouse cohort. In one group, mice experienced ovariectomy (OVX) to simulate an ovarian hormone-deficient state similar to surgical menopause, while the other group received Sham surgery as controls, to investigate the mechanistic role of ovarian hormone presence in driving immune dysregulation following acute WS exposure. Each experimental cohort followed a consecutive 2-day protocol with daily 4-h exposure intervals under two conditions: control HEPA-filtered air (FA) and acute WS to simulate an acute wildfire episode.

**Results:**

Metals analysis of WS particulate matter (PM) revealed significantly increased levels of ^63^Cu, ^182^W, ^208^Pb, and ^238^U, compared to filtered air (FA) controls, providing insights into the specific metal components most impacted by the changing dynamics of wildfire occurrences in the region. Male and female mice exhibited diverse patterns in lung mRNA cytokine expression following WS exposure, with males showing downregulation and females displaying upregulation, notably for IL-1β, TNF-α, CXCL-1, CCL-5, TGF-β, and IL-6. After acute WS exposure, there were notable differences in the responses of macrophages, neutrophils, and bronchoalveolar lavage (BAL) cytokines IL-10, IL-6, IL-1β, and TNF-α. Significant diverse alterations were observed in BAL cytokines, specifically IL-1β, IL-10, IL-6, and TNF-α, as well as in the populations of immune cells, such as macrophages and polymorphonuclear leukocytes, in both Sham and OVX mice, following acute WS exposure. These findings elucidated the profound influence of hormonal changes on inflammatory outcomes, delineating substantial sex-related differences in immune activation and revealing altered immune responses in OVX mice due to ovarian hormone deficiency. In addition, the flow cytometry analysis highlighted the complex interaction between OVX surgery, acute WS exposure, and their collective impact on immune cell populations within the hematopoietic bone marrow niche.

**Conclusions:**

In summary, both male and female mice, alongside females subjected to OVX and those who had sham surgery, exhibit significant variations in the expression of proinflammatory cytokines, chemokines, lung mRNA gene expression, and related functional networks linked to signaling pathways. These differences potentially act as mediators of sex-specific and hormonal influences in the systemic inflammatory response to acute WS exposure during a wildfire event. Understanding the regulatory roles of genes expressed differentially under environmental stressors holds considerable implications, aiding in identifying sex-specific therapeutic targets for addressing acute lung inflammation and injury.

**Supplementary Information:**

The online version contains supplementary material available at 10.1186/s12989-024-00587-5.

## Introduction

Wildfires have a substantial impact on air quality and ambient particulate matter (PM) [1]. Climate change has escalated the severity and frequency of wildfire incidents, which leads to an increase in the annual exposure of individuals to these occurrences [2]. Throughout 2020 wildfire events, densely populated and rural regions of the western United States, including areas within New Mexico [3], experienced unhealthy or hazardous air quality due to woodsmoke (WS)-derived ambient PM [4, 5]. Many investigations have established a correlation between WS and biomass exposure and increased mortality risk, as well as the aggravation of respiratory symptoms, notably in individuals with underlying health conditions like asthma, bronchitis, pneumonia, chronic obstructive pulmonary disease (COPD), and lung cancer [6, 7]. Woodsmoke contains a complex mixture of hundreds of chemical volatiles with toxic, carcinogenic, and irritant properties, as well as respirable PM that can infiltrate the airways and lungs [7, 8]. These exposures can generate downstream secondary blood-borne, biological molecules, including lipids, peptides and extracellular vesicles that drive toxic impacts in other organs [9, 10]. Consequently, this process induces lung inflammation, systemic inflammation, genotoxicity, and impacts populations of immune cells and innate immune system function in healthy individuals [7, 8]. Further investigations have indicated elevated glutathione concentrations in bronchoalveolar lavage fluid (BALF), accompanied by upper respiratory symptoms following exposure to WS [11]. Studies have revealed proinflammatory consequences at both systemic and respiratory levels, as evidenced by heightened neutrophil counts in peripheral blood and BALF in response to WS exposure [12]. Given the substantial diversity in global WS exposure, women are predominantly subjected to WS and biomass emissions indoors, often during cooking or heating activities, while men largely serve as first responders to household fires and wildfires [13]. Moreover, early exposure to WS has been shown to result in sex-specific alteration of immune response to infection by attenuating systemic TLR (toll-like receptor) responses, which leads to immune dysregulation and lung function decrements in adolescence [14]. Only female monkeys exposed to wildfire smoke showed a significant correlation between dynamic compliance and TLR ligand-induced cytokine synthesis, specifically for the induction of IL-6 by flagellin [14]. This highlights the necessity to recognize the potential impact of sex-related differences on the alterations of immune responses due to WS exposure.

**Fig. 1:**
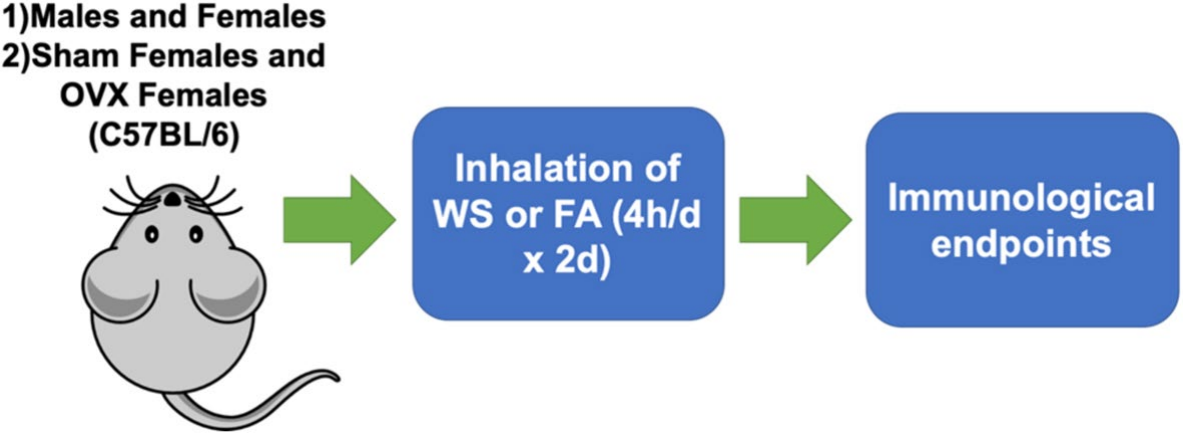
Graphical abstract. Male and female C57BL/6 mice, including Sham-operated and OVX females, experienced a 2-day exposure protocol with daily 4-h periods under acute woodsmoke (WS) from wildfires or control HEPA-filtered air (FA) conditions (*n* = *8* per group)

Biological sex plays a role in distinct immune responses owing to hormone receptors on immune cells and varying susceptibilities to different health challenges, with females typically showing more robust immune responses than males [15, 16]. Previous studies have suggested that sex hormones, including female hormones such as estrogen, human chorionic gonadotropin (hCG), and progesterone, and male hormone testosterone, play significant roles in modulating immune cell functionality [17–25]. These hormones have been implicated in influencing various aspects of immune responses, such as T-cell and B-cell activities, dendritic cell activation, and the development of T-cells. For example, estrogen is known to shape the development of T-cells and influence a wide range of functions, including CD4 + T-cell activities such as activation, cytokine production, differentiation, regulatory roles, and B cell stimulation for antibody production. Conversely, testosterone plays a pivotal role in stimulating type 1 T-helper cell function, crucial for effective cellular immune responses [24, 25]. In light of the existing evidence highlighting sex-based differences in the incidence and severity of lung health and diseases [26–28], there remains a critical need to address the knowledge gap concerning the influence of sex-specific factors and hormonal dynamics on the effects of WS exposure on respiratory health and immune responses. Hence, this study investigated potential variations in lung mRNA (messenger RNA) gene expression, BALF cytokine levels, and immune cell responses based on sex and hormonal status, therefore highlighting how acute WS exposure impacts lung health and immune regulation. Male and female C57BL/6 mice were used as in vivo models to elucidate the sex-specific responses to acute WS exposure. In a second set of investigations, female mice were either ovariectomized (OVX) or subjected to Sham surgery, with OVX representing ovarian hormone deficiency or post-surgical menopause, which enabled targeted investigation into the distinct roles of sex hormones, with a specific focus on understanding the impacts of OVX surgery, which entails the removal of ovarian hormones, or the estrogen- and progesterone-deficient state typical of surgical post-menopause, on lung mRNA gene expression, BALF and plasma cytokine levels, and immune cell populations following acute WS exposure. Quantitative flow cytometry analysis of bone marrow-derived cells from Sham and OVX groups to evaluate the impact of acute WS exposure on the hematopoietic immune cell population, while considering the potential impact of hormonal changes that resulted from OVX surgery. Each experimental group was exposed to a 2-day protocol, which consisted of daily 4-h intervals under both HEPA-filtered air (FA, the control setting) and acute WS conditions (Fig. 1). This systematic approach facilitated a comprehensive study of the effects that arise from sex and hormonal status in response to acute WS exposure.

## Results

### Metal analysis of WS particulates

**Fig. 2:**
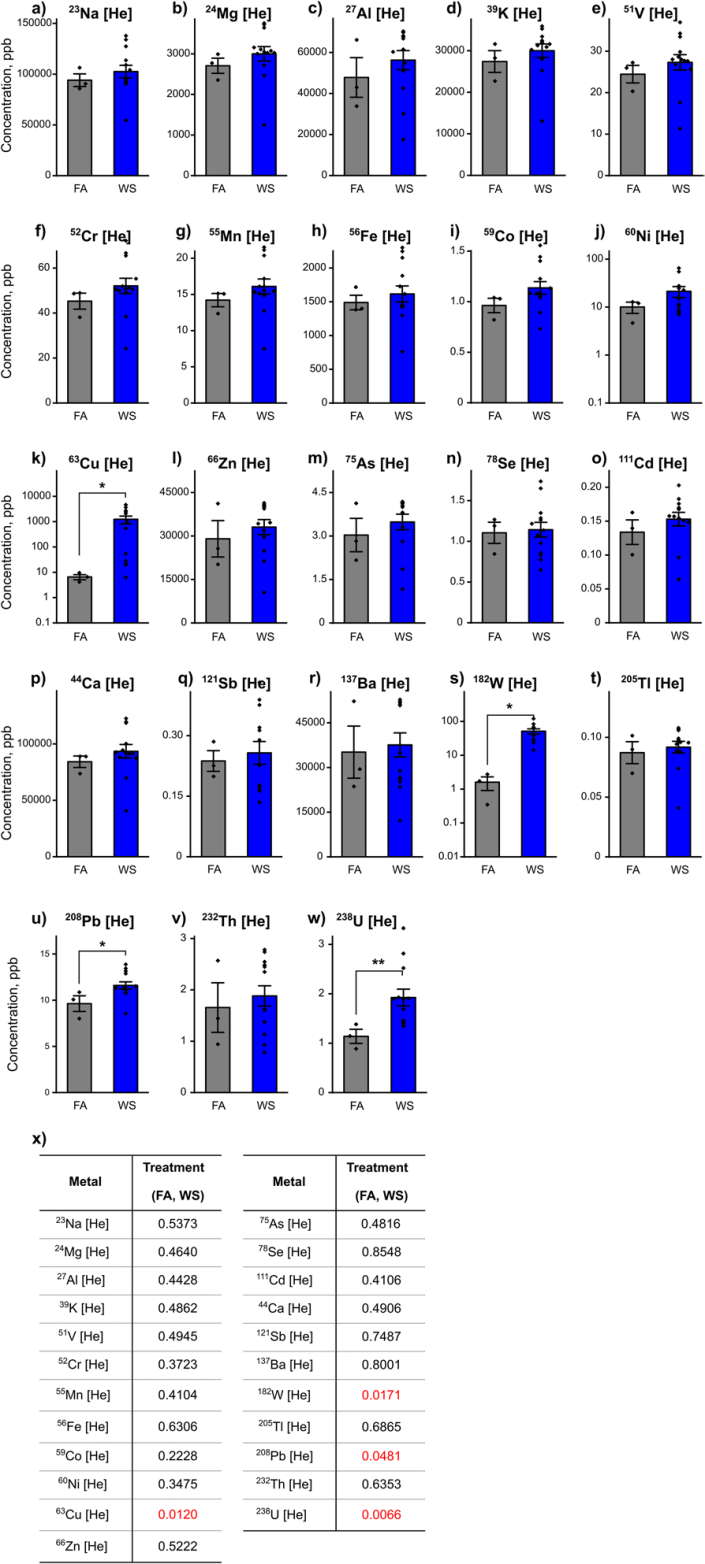
Metals analysis of particulate matter (PM) in FA and WS filters. The filters collected gravimetric samples post-inhalation exposure and were analyzed for trace metals (**a-w**) using inductively coupled plasma mass spectrometry (ICP-MS) methods in triplicate (*n* = *3*) for each group. All measurements were carried out under Helium [He] Mode to reduce potential interferences, and quality control samples were systematically integrated into the data acquisition and data analysis process to ensure data accuracy and reliability. Data were analyzed using the Mann–Whitney test, with (**x**) indicating the *P* value for the treatment variable (FA, WS) that influenced changes in metal concentrations (ppb), where *P* < *0.05* signified statistical significance. Asterisks denote statistically significant mean differences between groups: *P < 0.05; **P < *0.01*. Results are presented as mean ± SEM. The real-time measurements of concentrations (ppm, mg/m.^3^) of NO, CO, and O_3_ gases in the WS exposure chamber are reported in Supplemental Fig S1

Analysis of particulate matter (PM) metals indicated a consistent elevation in all detected metals within the acute WS-exposed group, in contrast to the control FA filters (Fig. 2a-w). This included statistically elevated levels of ^63^Cu, ^182^W, ^208^Pb, and ^238^U (*P* = *0.0120*, *P* = *0.0171*, *P* = *0.0481*, and *P* = *0.0066*, respectively, Fig. 2k,s,u,w,x). Albeit not statistically significant, ^23^Na, ^24^ Mg, ^27^Al, ^39^ K, ^51^ V, ^52^Cr, ^55^Mn, ^56^Fe, ^59^Co, ^60^Ni, ^66^Zn, ^75^As, ^78^Se, ^111^Cd, ^44^Ca, ^121^Sb, ^137^Ba, ^205^Tl, and ^232^Th demonstrated an observable pattern of increased concentration following acute WS exposure when compared to their respective FA controls (*P* = *0.5373*, *P* = *0.4640*, *P* = *0.4428*, *P* = *0.4862*, *P* = *0.4945*, *P* = *0.3723*, *P* = *0.4104*, *P* = *0.6306*, *P* = *0.2228*, *P* = *0.3475*, *P* = *0.5222*, *P* = *0.4816*, *P* = *0.8548*, *P* = *0.4106*, *P* = *0.4906*, *P* = *0.7487*, *P* = *0.8001*, *P* = *0.6865*, and *P* = *0.6353*, respectively, Fig. 2a-j,l-r,t,v,x). Before exposing male and female C57BL/6 mice, including Sham-operated and OVX females, to whole-body inhalation of WS, real-time monitoring of concentrations (ppm, mg/m^3^) of nitric oxide (NO), carbon monoxide (CO), and ozone (O_3_) gases in the WS exposure chamber was conducted. Woodsmoke exposure system operated overnight at a chamber temperature range of 21.3 – 21.5 °C, with samples collected every 10 min using the Grey Wolf TG501 gas sensor, and these data were presented in Supplemental Fig S1. Aerosizer size data for WS particulates, exhibited a mass median diameter (MMD) of the particles at 0.13 microns ± the standard deviation, with a geometric standard deviation of 1.4. The data derived from an average of 100 one-minute samples, with each sample constituting a one-minute measurement. This sampling methodology was used to enhance the precision and reliability of the particle size distribution analysis.

### Sex-dependent responses of lung mRNA gene expression, bronchoalveolar lavage cytokines, and immune cells to acute WS exposure

**Fig. 3:**
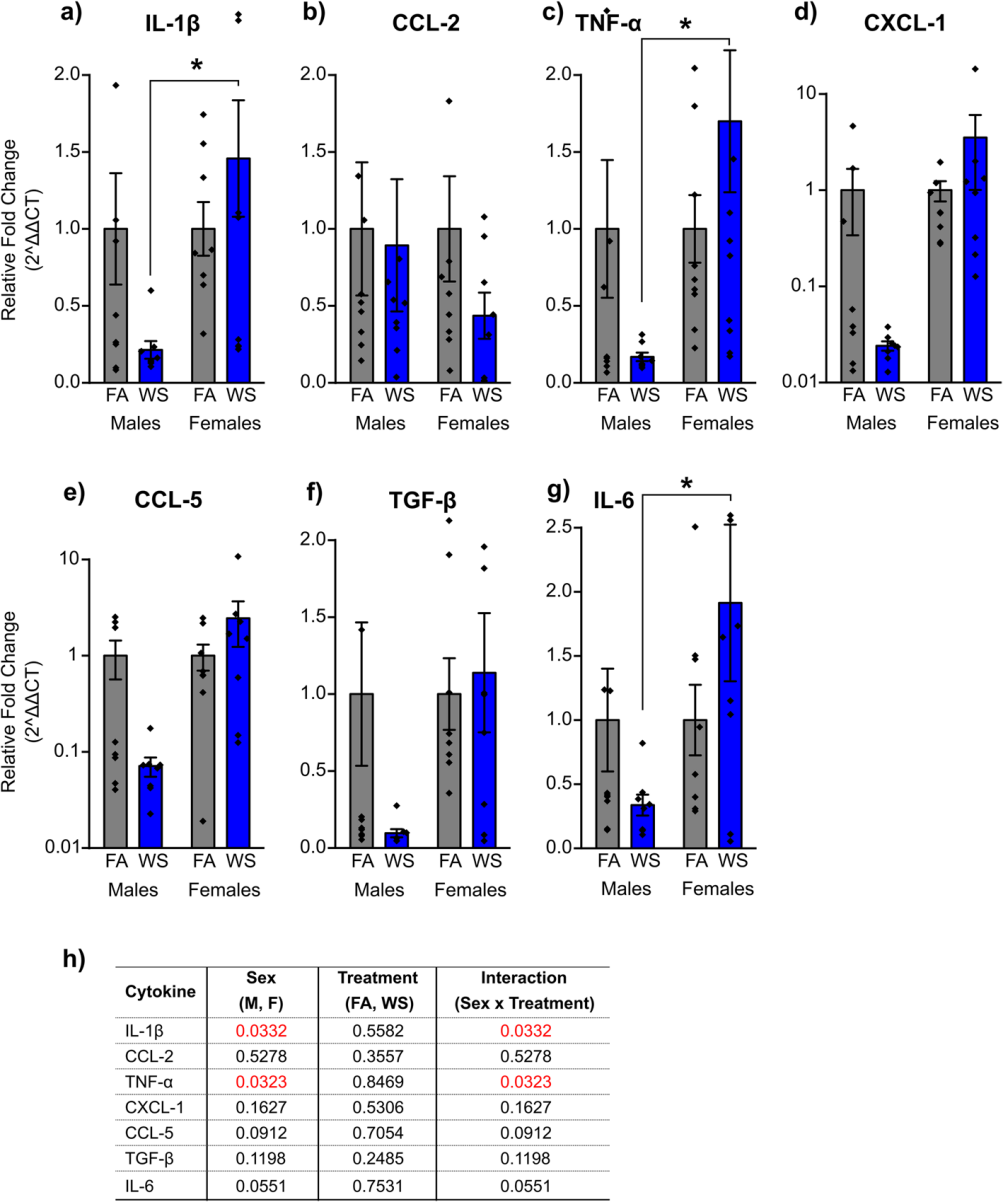
Sex-dependent (**a**-**g**) lung mRNA gene expression levels of cytokines in response to acute WS exposure. Lung mRNA relative gene expression levels demonstrated a response influenced by sex when exposed to WS. Data were analyzed using a two-way ANOVA, with **(h)** indicating the *P* value for each variable (sex, treatment, and interactions between sex and treatment) on lung mRNA genes, where *P* < *0.05* signified statistical significance. Asterisks denote statistically significant mean differences between groups, as identified by Tukey’s post-hoc test (*P < *0.05*). Data are presented as mean ± SEM. Each treatment group consisted of *n* = *8* mice. Mice across all groups were euthanized 24 h post-exposures

Woodsmoke exposure acute effects on sex-dependent responses including lung mRNA relative gene expression, bronchoalveolar lavage (BAL) cytokines, and immune cells from the hematopoietic bone marrow niche, were investigated. Assessment of lung mRNA cytokines in male and female C57BL/6 mice revealed diverse patterns in response to acute WS exposure (Fig. 3a-g). Following WS exposure, all lung mRNA gene expression levels of cytokines tested in this study (IL-1β, CCL-2, TNF-α, CXCL-1, CCL-5, TGF-β, and IL-6) exhibited a downregulated pattern in males compared to the cytokine levels in their respective FA controls, while females experienced upregulation levels, except for CCL-2. Only IL-1β and TNF-α lung mRNA gene expression levels were notably influenced by sex and the interaction between sex and treatment (*P* = *0.0332*, and *P* = *0.0323*, Fig. 3h). After WS exposure, males demonstrated reduced IL-1β lung mRNA gene expression, while females showed an increase, with a statistically significant difference in relative fold change (*P = *0.0182*, Fig. 3a), according to Tukey’s post-hoc test, which implies a sex-dependent response to IL-1β following acute WS exposure. Similar trends were evident for TNF-α and IL-6 responses (*P = *0.0175*, and *P = *0.0359*, respectively, Tukey’s post-hoc test, Fig. 3c,g), which suggests distinct sex-specific phenotype and that lung mRNA gene expression responses are impacted after acute WS exposure.

**Fig. 4:**
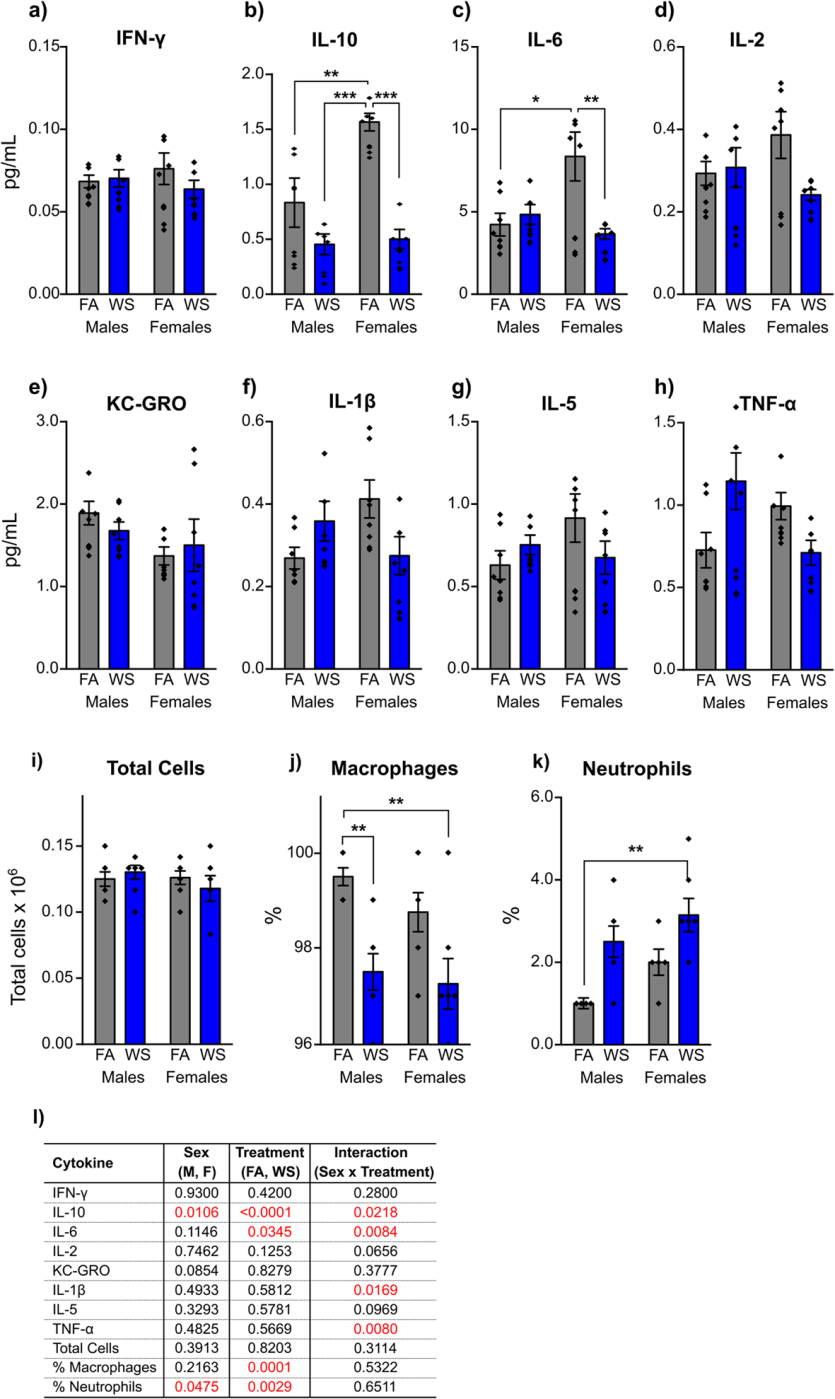
Effects of acute WS exposure on (**a**-**h**) bronchoalveolar lavage cytokine concentrations and (**i**-**k**) BALF immune cell responses in a sex-dependent manner. Data were analyzed using a two-way ANOVA, with (**l**) representing the corresponding *P* value for each variable (sex, treatment, and interactions between sex and treatment) that influenced changes in cytokines and BALF cell levels, where *P* < *0.05* signified statistical significance. Asterisks indicate significant mean differences among groups, as determined through multiple comparisons using Tukey’s post-hoc test: *P < 0.05; **P < 0.01; ***P < *0.001*. Results are presented as mean ± SEM. Each treatment group had *n* = *8* mice*.* Mice across all groups were euthanized 24 h post-exposures

There were no statistically significant changes in BAL IFN- γ, IL-2, KC-GRO and IL-5 (Fig. 4a,d,e,g). However, multiple cell types (total cells, macrophages and neutrophils, Fig. 4i-k), and cytokines tested in the BAL (IL-10, IL-6, IL-1β, and TNF-α, Fig. 4b,c,f,h) demonstrated differential responses following WS treatment. For example, the analysis revealed significant differences in various immune parameters among sex, treatment, and interaction groups (Fig. 4l). Specifically, from all of the tested BAL cytokines, only IL-10 concentrations were markedly impacted by a combination of factors, which included sex, treatment, and the interaction between sex and treatment (*P* = *0.0106*, *P* < *0.0001*, and *P* = *0.0218*, respectively, Fig. 4b,l). The concentration of IL-6 was significantly affected by treatment and the interaction between sex and treatment (*P* = *0.0345,* and *P* = *0.0084*, respectively, Fig. 4c,l). Both IL-10 (Fig. 4b) and IL-6 (Fig. 4c) concentrations exhibited the most significant differences among groups, which indicated pronounced immune dysregulation following acute WS exposure. Under FA control condition, females showed a notably higher IL-10 and IL-6 concentrations than males (**P = *0.0071*, and *P = *0.0210*, Tukey’s post-hoc test, Fig. 4b,c). Post-WS exposure, females exhibited a significant reduction in both IL-10 and IL-6 concentrations (***P = *0.0002*, and **P = *0.0084*, Tukey’s post-hoc test, Fig. 4b,c) when contrasted with their respective FA controls, which was not observed in males.

Acute WS exposure significantly influenced the percentages of macrophages and neutrophils (*P* = *0.0001,* and *P* = *0.0029*, Fig. 4j-l), while the percentage of neutrophils also impacted by sex (*P* = *0.0475*, Fig. 4k,l). Further examination revealed significant variations in the percentages of BAL macrophages in males after WS exposure compared to their FA control, while this trend was not evident in females (**P = *0.0067*, Tukey’s post-hoc test, Fig. 4j). Noteworthy differences were observed in both the percentages of macrophages and neutrophils in females following WS exposure, in contrast to the FA controls in males (**P = *0.0021,* and **P = *0.0068*, respectively, Tukey’s post-hoc test, Fig. 4j,k). All cytokines detected in the plasma showed no significant differences among groups (data not shown), which was consistent with prior pre-clinical literature on air pollution [29, 30].

### Ovariectomy-dependent effects on lung mRNA gene expression, cytokines, and immune cells in response to acute WS exposure

**Fig. 5:**
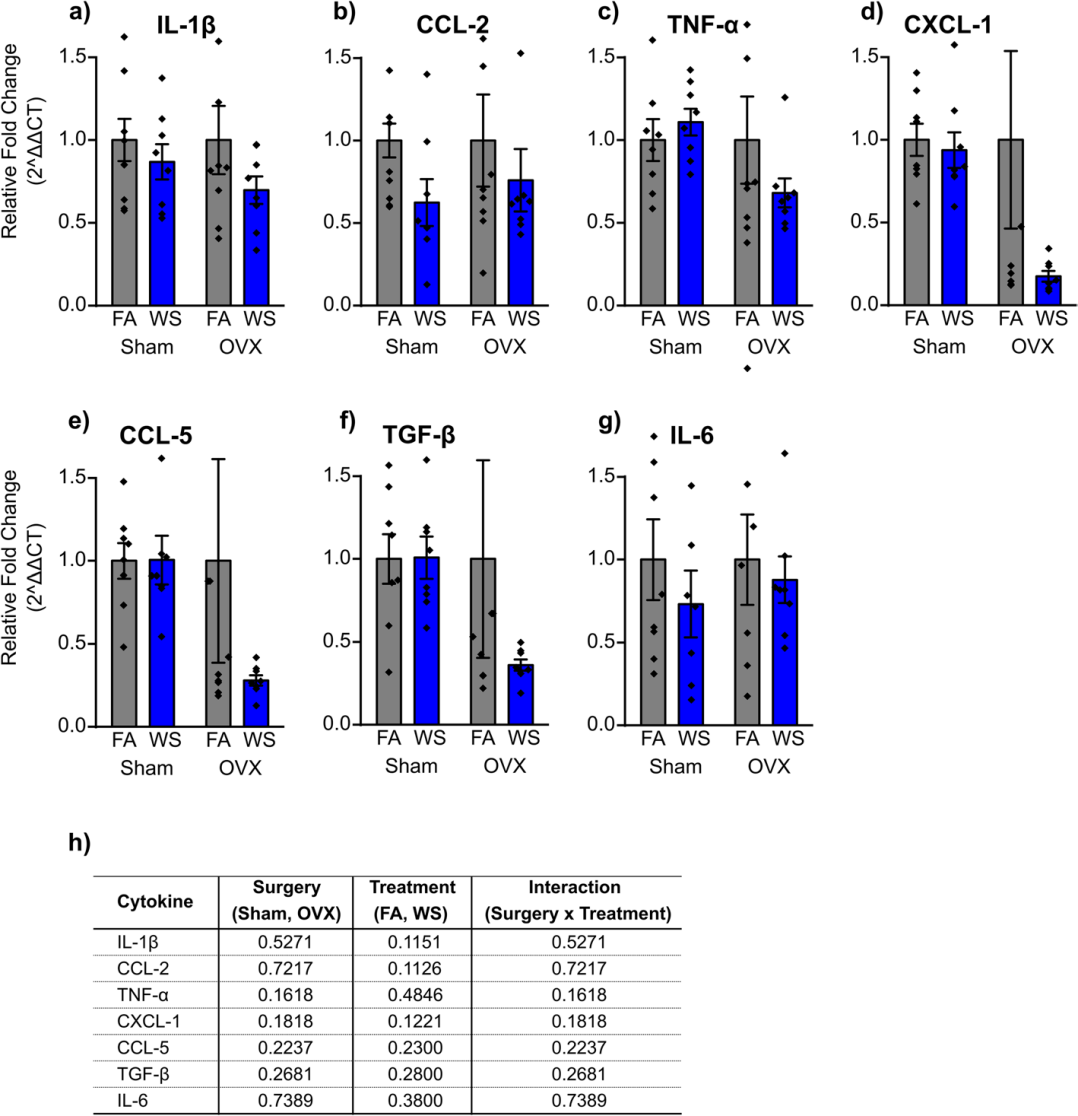
Lung mRNA gene expression levels following Sham or OVX surgery and FA or WS exposure (**a-g**). Analysis was conducted using a two-way ANOVA, with (**h**) representing the *P* value for each variable (surgery, treatment, and interactions between surgery and treatment) in relation to gene expression, where *P* < *0.05* indicated statistical significance. Data are presented as mean ± SEM. Each treatment group consisted of *n* = *8* mice. Mice across all groups were euthanized 24 h post-exposures

The data demonstrated that neither ovariectomy surgery, exposure treatment, nor the interaction between OVX surgery and treatment significantly altered the expression of all lung mRNA genes examined (IL-1β, CCL-2, TNF-α, CXCL-1, CCL-5, TGF-β, and IL-6, as shown in Fig. 5a-h). After WS exposure in OVX mice, the cytokine levels of all lung mRNA decreased compared to their corresponding FA controls, and they were also lower than the cytokine levels in Sham mice, except for CCL-2 and IL-6, albeit not significantly. Overall, our results suggest that neither WS-exposure nor ovarian hormone presence had any statistically significant impact on lung gene expression.

**Fig. 6:**
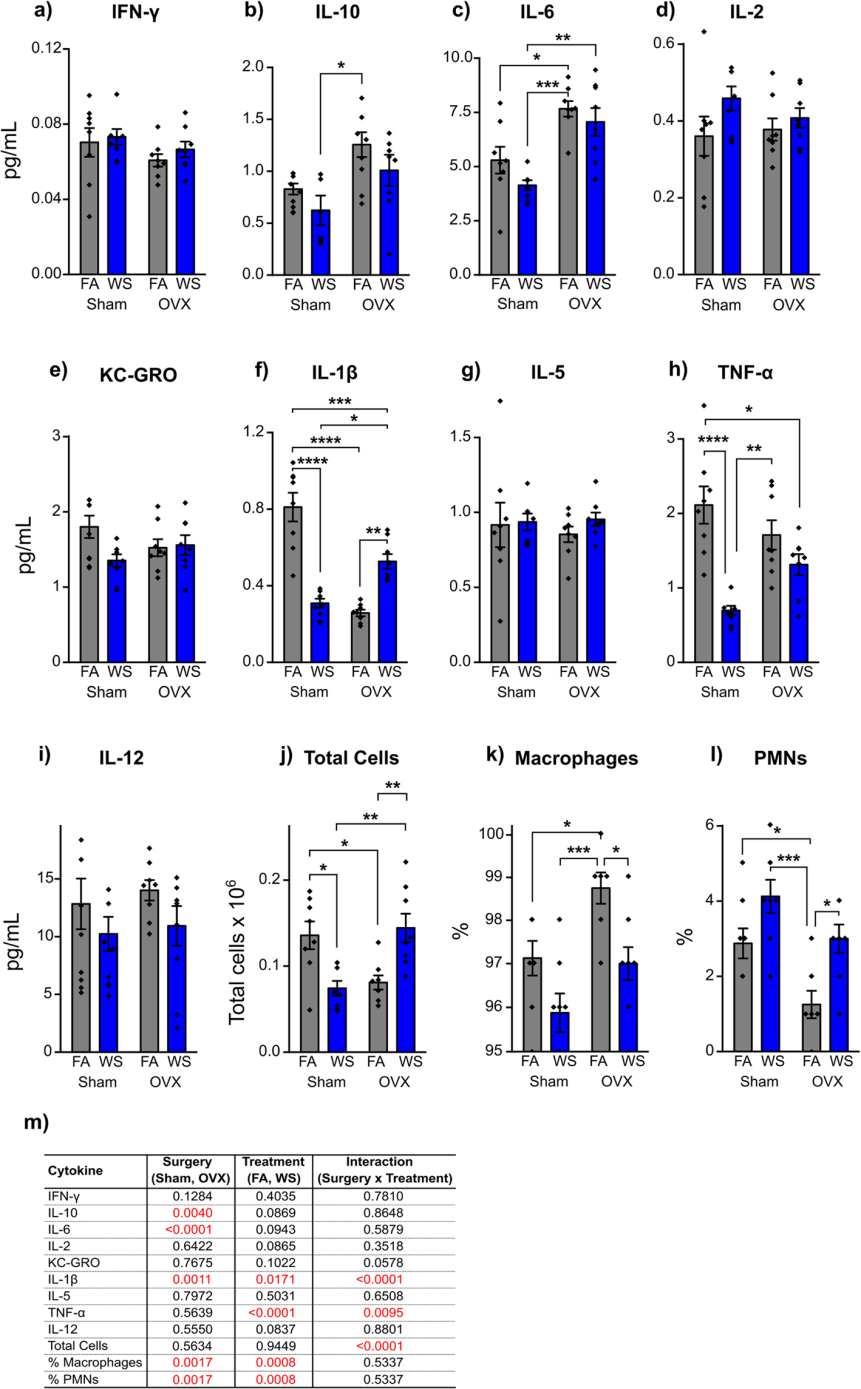
Acute WS exposure-induced changes in (**a-i**) bronchoalveolar lavage cytokine concentrations and (**j-l**) immune cell responses following Sham or OVX surgery. Analysis involved a two-way ANOVA, where (**m**) represented the corresponding *P* value for each variable (surgery, treatment, and interactions between surgery and treatment) that contributed to the alterations in cytokines and BALF cell levels, with *P* < *0.05* signifying statistical significance. Asterisks indicated significant mean differences among groups, as determined through multiple comparisons using Tukey’s post-hoc test: *P < 0.05; **P < 0.01; ***P < 0.001; ****P < *0.0001*. Results are presented as mean ± SEM. Each treatment group had a total of *n* = *8* mice. Mice across all groups were euthanized 24 h post-exposures

The analysis of BAL cytokines in Sham and OVX mice disclosed diverse alterations in concentrations of lavage cytokines and percentage of immune cell populations in response to acute WS exposure (Fig. 6a-l). Particularly, only IL-1β was significantly influenced by various factors, which involved ovariectomy surgery (*P* = *0.0011*, Fig. 6f,m), treatment (*P* = *0.0171*, Fig. 6f,m), and the interaction between surgery and treatment (*P* < *0.0001*, Fig. 6f,m). Both IL-10 and IL-6 cytokines experienced notable impacts from ovariectomy surgery (*P* = *0.0040* and *P* < *0.0001*, respectively, Fig. 6b,c,m), while TNF-α showed significant alterations due to treatment (*P* < *0.0001*) and the interaction between surgery and treatment (*P* = *0.0095*, Fig. 6h,m). The data demonstrated that OVX mice exhibited notably increased concentrations of IL-6 under both FA and acute WS exposure (*P = *0.0116*, **P = *0.0023*, respectively, Tukey’s post-hoc test, Fig. 6c). Following WS exposure, concentration of IL-1β was prominently reduced in Sham mice (****P < *0.0001*, Tukey’s post-hoc test), whereas in OVX mice, it was significantly upregulated (**P = *0.0013*, Tukey’s post-hoc test) compared to their respective FA controls (Fig. 6f). The difference in IL-6 and IL-1β concentrations between Sham and OVX mice, whether under FA control (*P = *0.0116* and ****P < *0.0001*, respectively, Tukey’s post-hoc test) or after WS exposure (**P = *0.0023*, and *P = *0.0132*, respectively, Tukey’s post-hoc test), was statistically significant (Fig. 6c,f). Moreover, notable reductions in TNF-α concentration were noted in Sham mice following WS exposure (****P < *0.0001*, Tukey’s post-hoc test), whereas no such effect was observed in OVX mice (Fig. 6h).

Concerning BAL immune cells, both percentages of macrophages and polymorphonuclear leukocytes (PMNs) experienced pronounced effects due to ovariectomy surgery (*P* = *0.0017*, Fig. 6k-m) and treatment factors (*P* = *0.0008*, Fig. 6k-m). In response to acute WS exposure, both Sham and OVX mice showed reduced percentages of macrophages and elevated percentages of PMNs compared to their respective FA controls, however, a significant difference was evident solely in the OVX mice group (*P = *0.0205,* Tukey’s post-hoc test, Fig. 6k,l).

### Ovariectomy-dependent response of bone marrow-derived hematopoietic cells following acute WS exposure

**Fig. 7:**
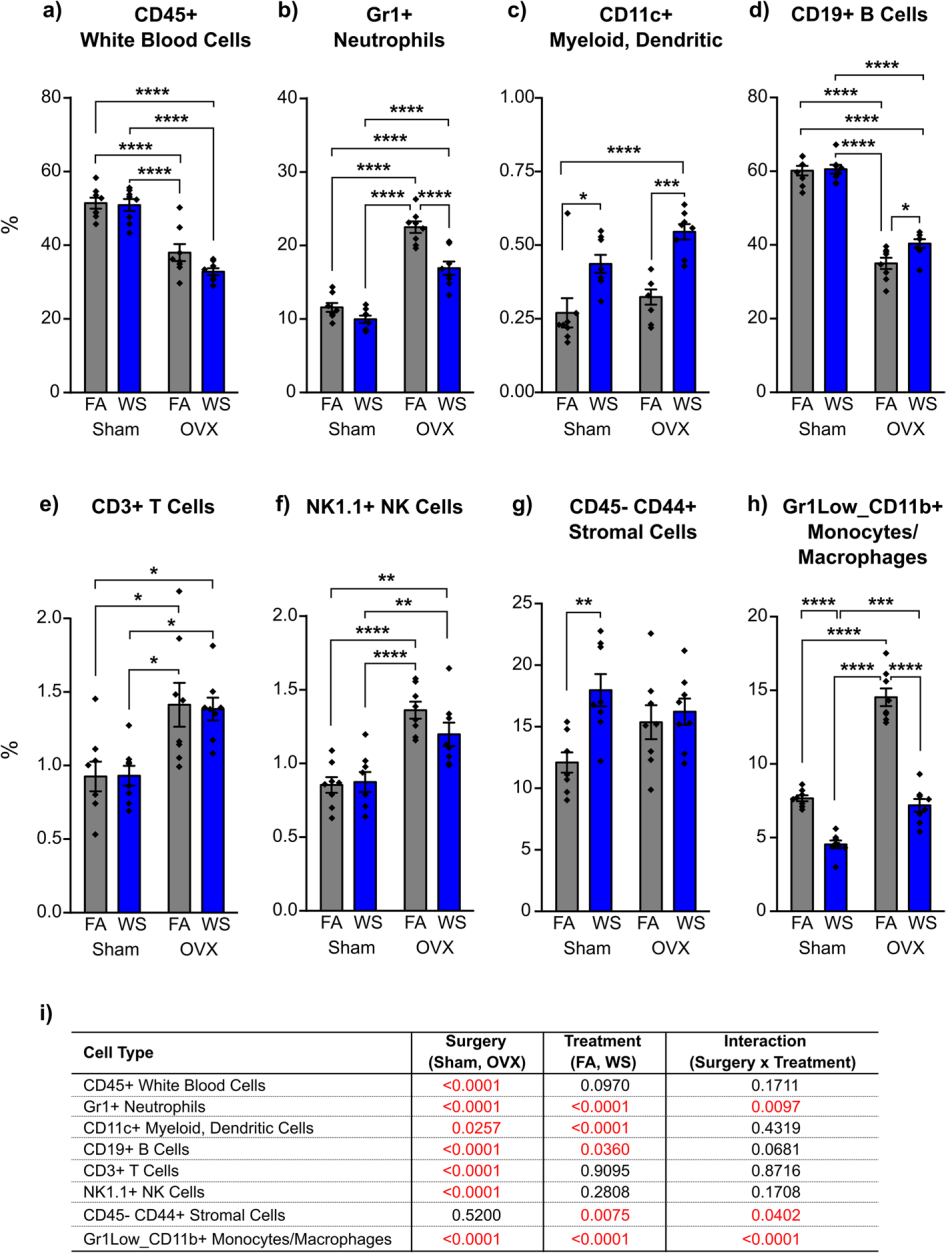
Characterization of (**a-h**) bone marrow-derived cellular subpopulations in ovarian hormone deficiency (OVX) and Sham mice following acute WS exposure. Data analysis was conducted through a two-way ANOVA, where (**i**) represented the respective *P* value for each variable (surgery, treatment, and interactions between surgery and treatment), elucidating their contributions to the variations in bone marrow-derived cellular subpopulations, with *P* < 0.05 indicated statistical significance. Asterisks highlight significant mean differences between groups, discerned by using Tukey’s post-hoc test for multiple comparisons: *P < 0.05; **P < 0.01; ***P < 0.001; ****P < 0.0001. Results are presented as the mean ± SEM. Cellular subpopulations were quantified using flow cytometry of total bone marrow cells, as shown in Supplemental Fig. S2, stained with different cell surface markers listed in Table 1. Each treatment group consisted of *n* = 8 mice. Mice across all groups were euthanized 24 h post-exposures

Ovariectomization led to a notable reduction in the percentage of total white blood cells in the bone marrow (CD45 + cells) in comparison to Sham mice, both under FA control and acute WS exposure (****P < *0.0001* for OVX-FA versus Sham-FA, OVX-FA versus Sham-WS, OVX-WS versus Sham-FA, and for OVX-WS versus Sham-WS*,* Tukey’s post-hoc test, Fig. 7a, and Supplemental Fig S2a), which signified a considerable impact of ovariectomy surgery on the amount of CD45 + cells in the bone marrow (*P* < *0.0001*, Fig. 7i).

Subsequently, following WS exposure, the percentage of neutrophils (Gr1 + cells) was reduced in OVX mice compared to FA controls (****P < *0.0001* for OVX-WS versus OVX-FA, Tukey’s post-hoc test, Fig. 7b, and Supplemental Fig S2b), whereas this change was not apparent in Sham mice. Moreover, OVX mice had a substantial increase in the percentage of neutrophils (Gr1 + cells) relative to Sham mice, evident both under FA control and following acute WS exposure (****P < *0.0001* for OVX-FA versus Sham-FA, OVX-FA versus Sham-WS, OVX-WS versus Sham-FA, and for OVX-WS versus OVX-FA, Tukey’s post-hoc test, Fig. 7b, and Supplemental Fig S2b), suggesting an influence of various factors, including ovariectomy surgery and treatment (*P* < *0.0001*, Fig. 7i), as well as the interaction between surgery and treatment on the percentage of Gr1 + cells (*P* = *0.0097*, respectively, Fig. 7i).

Upon acute WS exposure, the percentage of myeloid cells, including dendritic cells (CD11c + cells) was significantly increased in both Sham and OVX mice compared to their respective FA controls (*P = *0.0102* for Sham-WS versus Sham-FA, and ***P = *0.0005* for OVX-WS versus OVX-FA, respectively, Tukey’s post-hoc test, Fig. 7c, and Supplemental Fig S2c), indicating the impact of both ovariectomy surgery (*P* = *0.0257*) and treatment (*P* < *0.0001*) on the percentage of CD11c + cells (Fig. 7i).

Simultaneously, the percentage of B cells (CD19 + cells) following acute WS exposure in OVX mice was increased compared to FA controls (*P = *0.0342* for OVX-WS versus OVX-FA, Tukey’s post-hoc test, Fig. 7d, and Supplemental Fig S2e), whereas this change was not discernible in the Sham mice. Precisely, there was a significant attenuation of the percentage of B cells (CD19 + cells) in OVX mice compared to Sham mice, evident under both FA control and acute WS exposure conditions (****P < *0.0001* for OVX-FA versus Sham-FA, OVX-FA versus Sham-WS, OVX-WS versus Sham-FA, and for OVX-WS versus Sham-WS, Tukey’s post-hoc test, Fig. 7d, and Supplemental Fig S2e), highlighting the substantial influence of both ovariectomy surgery (*P* < *0.0001*) and treatment (*P* = *0.0360*) on the alterations in the percentage of CD19 + cells (Fig. 7i).

Furthermore, the percentages of T cells (CD3 + cells) and NK cells (NK1.1 + cells) showed a comparable pattern with a significant increase in the percentages of these cell types in OVX mice under both FA control and acute WS exposure conditions when compared with Sham mice (T cells: *P = *0.0123* for OVX-FA versus Sham-FA, *P = *0.0134* for OVX-FA versus Sham-WS, *P = *0.0198* for OVX-WS versus Sham-FA, and *P = *0.0214* for OVX-WS versus Sham-WS, Fig. 7e, and Supplemental Fig S2f, as well as NK cells: ****P < *0.0001* for OVX-FA versus Sham-FA and for OVX-FA versus Sham-WS, **P = *0.0047* for OVX-WS versus Sham-FA, and **P = *0.0080* for OVX-WS versus Sham-WS, Tukey’s post-hoc test, Fig. 7f, and Supplemental Fig S2g), which signifies the role of ovariectomy surgery on these immune cell populations (*P* < *0.0001* for both T cells and NK cells, Fig. 7i).

Regarding stromal cells, there was a notable increase in the percentage of CD45-/CD44 + cells following acute WS exposure in Sham mice (**P = *0.0069* for Sham-WS versus Sham-FA, Tukey’s post-hoc test, Fig. 7g, and Supplemental Fig S2d), whereas this change was absent in OVX mice, validating the impact of both treatment (*P* = *0.0075*) and the interaction between surgery and treatment (*P* = *0.0402*) on changes in stromal cells (Fig. 7i).

In both Sham and OVX mice, the percentage of monocytes/macrophages (Gr1low/CD11b + cells) was decreased following acute WS exposure, in relation to FA controls for each group (****P < *0.0001* for Sham-WS versus Sham-FA and for OVX-WS versus OVX-FA, Tukey’s post-hoc test, Fig. 7h, and Supplemental Fig S2h). In the OVX mice, the percentage of monocytes/macrophages under both FA control and acute WS exposure conditions were significantly increased compared to Sham mice (****P < *0.0001* for OVX-FA versus Sham-FA, OVX-FA versus Sham-WS, and for OVX-WS versus Sham-WS, Tukey’s post-hoc test, Fig. 7h, and Supplemental Fig S2h), which affirms the combined impact of ovariectomy surgery, treatment, and the statistical interaction between surgery and treatment on the population of monocytes/macrophages, (*P* < *0.0001*, Fig. 7i).

## Discussion

In recent years, the United States has experienced a significant increase in the frequency and severity of acute wildfire events, posing substantial challenges to public safety [1–3]. Given this situation, it is essential to investigate the intricate association between these escalating wildfires and their potential implications for immunological and inflammatory outcomes. Furthermore, exploration of how systemic immunological responses, including their potential dependence on sex and the presence of ovarian hormones, are affected by acute woodsmoke (WS) exposure. Cytokine analysis investigated potential variations in lung mRNA gene expressions and levels of cytokines in BAL, while sex differences and ovarian hormonal factors were considered, to provide in-depth insights into the impact of acute WS exposure on lung health and immune system regulation.

Significantly upregulated levels of IL-1β, TNF-α, and IL-6 mRNA gene expression in the lung tissue were observed in females compared to males following acute WS exposure (Fig. 3a,c,g). The results indicate a more robust and pronounced inflammatory response in females in response to this environmental stimulus [31], as evidenced by a two-fold increase in the levels of these proinflammatory cytokines compared to males (Fig. 3a,c,g), which highlight substantial sex-related differences in immune activation following WS exposure and suggest that based on these data, males exhibit an immunosuppressive phenotype and females a proinflammatory response in the lungs, specifically IL-1β, TNF-α, and IL-6 mRNA gene expression. These genes have been associated with chronic inflammation and play a significant role in lung inflammatory diseases like lung cancer by recruiting and activating leukocytes and directly contributing to lung inflammation [32]. The activation of these IL-1β, TNF, and IL6 genes in individuals following short-term (acute) exposure, such as acute WS exposure from a wildfire event, can lead to a microenvironment with significantly altered or enhanced intensity of the inflammatory response [31], thereby increasing the risk of developing lung diseases [33]. The significant downregulation of proinflammatory markers, IL-1β, TNF-α, and IL-6, in males compared to females after acute WS exposure should not be construed as an absence of response but rather as a distinct immune modulation, which indicates that males rely on different immunoregulatory mechanisms [34]. These findings suggest a potential sexual dimorphism in the mechanisms governing inflammatory responses to environmental exposures, like acute WS exposure [34]. Some cytokines involved in lung mRNA gene expression exhibited no significant sex-related differences in their response to acute WS exposure. These observations raised several potential implications: (i) immune responses related to lung mRNA gene expression of CCL-2, CXCL-1, CCL5, and TGF-β appeared comparable between sexes following acute WS exposure, possibly due to shared regulatory mechanisms, as there was no observed correlation between sex, or other factors that might have mitigated sex-related disparities [35]; (ii) CCL-2, CXCL-1, CCL5, and TGF-β cytokines in the lung tissue had distinct roles or regulatory patterns compared to IL-1β, TNF-α, and IL-6, which suggests the complexity of individual responses to environmental stimuli like acute WS exposure during wildfires, influenced by sex-specific hormonal and genetic factors [36, 37]; (iii) both sexes demonstrated the capacity to mount effective immune responses to acute WS exposure concerning these specific cytokines, and this balanced immune response can be crucial for overall health and the ability to respond to environmental challenges.

Acute WS exposure affected the concentrations of BAL cytokines differently, with a notable decrease in IL-10 and IL-6 in female mice (Fig. 4b,c), while in males, the concentrations of all analyzed BAL cytokines, IFN-γ, IL-10, IL-6, IL-2, KC-GRO, IL-1β, IL-5, and TNF-α, remained significantly unchanged compared to FA controls (Fig. 4a-h). These responses imply that females exhibited a more active and robust immune response in the BAL, with significant reductions in IL-10 and IL-6 concentrations (Fig. 4b,c), which indicates a possible strong regulatory response to restore immune balance. The significant changes in lung mRNA gene expression between males and females, specifically those related to IL-1β, TNF-α, and IL-6 (Fig. 3a,c,g), exhibited no correlation with the alterations in the concentrations of IL-1β, TNF-α, and IL-6 cytokines observed in BAL following acute WS exposure (Fig. 4c,f,h). Interestingly, under control FA conditions, both IL-10 and IL-6 concentrations in BAL were significantly higher in females compared to males (Fig. 4b,c). These results illuminate the role of pro-inflammatory cytokines in females, particularly IL-6, independent of TNF-α, in potentially stimulating the increased production of anti-inflammatory molecules, such as IL-10 [38], in response to acute WS exposure. This finding aligns with the outcome of OVX surgery, which statistically impacts IL-10 cytokines in response to acute WS exposure (Fig. 6b,m), suggesting that endocrine profiles might potentially impact IL-10 mechanisms, specifically [39]. This regulatory mechanism serves to mitigate lung inflammation and maintain immune homeostasis [38, 40]. Simultaneously, the female immune system actively responds to acute WS exposure, resolves lung inflammation, and consequently leads to a significant decrease in the concentrations of IL-6 and IL-10 BAL cytokines (Fig. 4b,c) [40]. Moreover, these findings elucidate the influence of sex hormones, such as progesterone or estrogen, on the upregulation of IL-10 and IL-6 cytokines and inflammatory mediators in the lung [20], which suggests that females may possess a more effective defense against respiratory infections or inflammation in the lungs under specific conditions [20]. There was a significant difference in the percentage of macrophages in male mice after acute WS exposure compared to the control group exposed to FA, while such differences were not observed in females (Fig. 4j), which implies a distinctive susceptibility of male mice to the effects of acute WS exposure on macrophage populations and a sex-specific response to acute WS exposure. Wood combustion generates a highly complex mixture of carcinogenic chemical compounds. In the lungs, alveolar macrophages, as a subpopulation of immune cells, play central roles in the innate immune system and are responsible for the removal of pathogens and non-pathogens in the lung, particularly the clearance of inhaled PM [41]. In addition, macrophages from BAL exposed to acute WS generate free radicals, exhibit an inflammatory response, and induce TNF-α and other cytokine release [42]. These findings highlight the complexity of the immune response to acute WS exposure and underscore the potential influence of sex-specific responses.

While our study did not detect sex-based differences in plasma cytokine levels, future investigations should explore the impact of sex hormones on WS-induced cytokine regulation, both locally and systemically, given the observed differences in cytokine levels between lung tissue and plasma. Air pollution exposures typically do not alter plasma cytokine levels, but secondary systemic effects involving circulating factors such as peptides, extracellular vesicles, and serum-borne byproducts, distinct from canonical cytokines, could play a pivotal role [10, 43]. As the lungs and pulmonary system represent the primary target organs for inhaled toxicants, the most significant impact was observed [10, 43]. However, examinations of terminally differentiated cells within the bone marrow niche remain underexplored, despite potential interactions with these secondary factors in that specific microenvironment.

Numerous lung disorders and cancers exhibit sex disparities in their prevalence, severity, or outcome, for instance, inflammatory lung diseases like asthma [44, 45], which distinctly reveal a pronounced sexual dimorphism throughout the lifespan [44, 45]. A noteworthy observation is that while childhood asthma tends to be diagnosed more often in male children than female children, studies within adult populations often reveal less favorable lung health outcomes for women compared to men, which indicates a potential role of sex hormones in mediating these effects, that involve menstruation, pregnancy, the menopausal phase, and hormone-based treatments [44, 45]. Additionally, it has been postulated that female sex hormones function as physiological regulators of lung function and immunity in women, by way of inflammatory gene expression regulation [31, 46, 47]. Surprisingly, despite the pronounced hormonal changes induced by OVX, there were no statistically significant effects on lung mRNA expression levels of any of the inflammatory molecules (cytokines: IL-1β, TNF-α, TGF-β, and IL-6, and chemokines: CCL-2, CXCL-1, CCL-5) at the time points tested when comparing OVX and Sham groups after acute WS exposure, nor when comparing OVX and Sham groups after WS exposure to their respective FA control groups (Fig. 5a-h). While some prior research suggests that ovarian hormone deficiency triggers inflammation, others present contrasting views [48]. For instance, OVX had no effect on mRNA expression of the cytokines IL-1α, IL-1β, IL-6, and TNF-α in the bone marrow and BAL supernatants [48]. Conversely, in the serum of women who had undergone a complete hysterectomy and bilateral oophorectomy [49], within bone marrow cultures of estrogen-deficient females and rodents [50, 51], and in the bone marrow supernatants collected from mice two weeks post-OVX [52], an increase in cytokine levels was detected. Previous findings and the results of the current study suggest that other cytokines and chemokines might be involved under estrogen-deficient conditions when the immune system faces greater challenges, as in the case of multiple traumas, hemorrhage, or sepsis. In addition, our study did not explore subpopulations of lung cell types, which may demonstrate more sensitivity to ovarian hormone deficiency.

The analysis of BAL cytokine concentrations and immune cell populations in response to acute WS exposure yielded insights into the potential influence of ovarian hormone deficiency. It was observed that concentrations of the pro-inflammatory cytokine IL-6 significantly increased in the BAL of OVX mice when compared to Sham-operated counterparts, both under FA and after acute exposure to WS (Fig. 6c). Thus, it is plausible that the observed exaggerated inflammatory response in OVX-mouse lungs could be attributed to the absence of estrogen, which is known to exhibit both anti-inflammatory and pro-inflammatory properties [53, 54]. Acute WS exposure from wildfires contains various harmful particles and chemicals, including those that can irritate and inflame the respiratory system. The higher IL-6 concentrations in OVX mice after acute WS exposure indicate that the combination of hormonal changes and environmental exposure can demonstrate a statistically significant interaction on lung inflammatory outcomes. Upregulation of IL-6 concentrations is associated with increased inflammation, which can contribute to respiratory diseases and other health issues [55]. In contrast, following acute WS exposure, IL-1β concentrations significantly decreased in Sham-operated mice but substantially increased in OVX mice compared to their respective FA controls (Fig. 6f). This indicates that OVX disrupted the usual immune response observed in Sham-operated mice, leading to a dysregulated IL-1β response to environmental stimuli. The contrasting responses in IL-1β concentrations between Sham and OVX mice after acute WS exposure underscore the intricate interplay between hormonal status and inflammation. Furthermore, the statistical significance of the differences in IL-6 and IL-1β concentrations between Sham and OVX mice, both under FA control and after acute WS exposure, underscores the robustness and reliability of these observations. These significant differences strongly suggest that the hormonal changes associated with OVX have a substantial impact on lung inflammation. The role of estrogen to modulate IL-1β production aligns with prior research that has shown an elevated IL-1β production in ex vivo cultures of unstimulated macrophages in the presence of estrogen deficiency [56–58], as well as in vivo following lung injury induced by carrageenan [59]. In its role regulating chemokine and cell adhesion molecule expression during lung injury, IL-1β exerts a potent anti-apoptotic effect in various cell types, including neutrophils and epithelial cells [60, 61]. Apoptosis, a controlled cell death process, plays a pivotal role in tissue remodeling during repair, yet its relevance in acute lung injury remains a topic of debate [62]. In this current study, the notable reduction in IL-1β production observed in Sham mice following acute WS exposure (Fig. 6f) suggests a potential beneficial facet of estrogen presence. This hypothesis is in line with prior research suggesting that estrogen can trigger apoptosis in macrophages by interacting with the Fas ligand promoter [63]. A deeper exploration of the mechanisms through which estrogen mediates apoptosis and its relationship with specific cell types could advance understanding of estrogen in acute lung inflammation [64]. Notable reductions in TNF-α concentration were observed in Sham-operated mice following acute WS exposure, while OVX mice did not exhibit such a reduction (Fig. 6h). This discrepancy indicates that the absence of ovarian hormones in OVX mice may alter the usual immune system response to acute WS exposure, potentially resulting in less pronounced decreases in TNF-α concentrations. The presented data highlight the significant impact of OVX surgery and treatment factors on immune cells, including macrophages (Fig. 6k) and polymorphonuclear leukocytes (Fig. 6l). When these mice were exposed to acute WS, both Sham-operated and OVX groups exhibited increased percentages of PMNs in comparison to their respective FA control groups. However, a crucial distinction emerged as the elevation in percentages of PMNs was statistically significant in OVX mice but not in Sham-operated mice, which indicates a more pronounced effect of OVX on PMN percentages in response to acute WS exposure. Furthermore, notable differences in percentages of macrophages were observed between the FA-control and acute-WS exposure groups in OVX mice, whereas no such distinctions were evident in the Sham group, which suggests that OVX has an impact on the response of lung macrophages to WS exposure. In the context of lung injury, IL-1β serves as an early mediator of acute lung injury and is linked to increased production of CXC chemokines in the BALF, which are necessary for attracting PMNs into the lung [64]. When IL-1β concentrations and the percentage of PMNs significantly increased after acute WS exposure in OVX mice, it may indicates that the estrogen depletion rendered OVX mice more susceptible to inflammation and tissue damage in acute lung injury induced by acute WS exposure. This mechanism potentially contributes to the heightened accumulation of PMNs and exacerbated lung injury observed in OVX mice. Therefore, these findings collectively imply that post-surgical menopause may induce a more vulnerable to the adverse effects of environmental pollutants, such as acute WS exposure. In Fig. 6, OVX females exhibited a significantly increased neutrophilia in the lung following WS-exposure, while Sham females showed a non-significant rise. This suggests that neutrophilic infiltration in the lung is more pronounced in OVX mice after acute WS-exposure. While the data indicate a significant influence of ovarian hormones on neutrophil presence in the lungs and hematopoietic niche post-exposure, drawing definitive biological conclusions remains premature, and heightened responsiveness to WS could potentially impact health-related endpoints, including susceptibility to infection.

The data elucidate the intricate interplay involving OVX surgery, acute WS exposure, and their cumulative effects on immune cell populations (Fig. 7, and Supplemental Fig S2) in the hematopoietic bone marrow niche. Notably, OVX surgery resulted in a significant reduction in the percentages of white blood cells (CD45 + cells) compared to Sham-operated mice, regardless of exposure conditions. Moreover, OVX mice exhibited a distinct response in the percentage of neutrophils (Gr1 + cells) following acute WS exposure, showing both a decrease compared to FA controls and an increase compared to Sham mice. This differential response suggests the influence of multiple factors, including surgery, treatment, and their interactions, on the bone-marrow derived neutrophil population. One possible interpretation of these data is that the hormonal alterations accompanying OVX may exert a significant influence on the immune system, leading to this neutrophil response. The pronounced decrease in the percentage of neutrophils in OVX mice following acute WS exposure, when compared to FA control, may potentially suggest a compromised initial immune response, possibly influenced by estrogen and progesterone depletion. This reduced immune activity could render OVX mice more susceptible to certain infections or inflammations, [65, 66], given the critical role of neutrophils in regulating adaptive immune responses [67]. Conversely, the marked increase in the percentage of neutrophils in OVX mice, in comparison to Sham-operated mice, whether in the FA control group or following acute WS exposure, might suggest an exaggerated response, which, although advantageous in specific contexts [68], could also have detrimental effects [69].

Myeloid cells, including dendritic cells (CD11c + cells) showed a significant increase in both Sham and OVX mice after acute WS exposure, emphasizing the impact of these factors on this immune cell population. Additionally, B cells (CD19 + cells) in OVX mice exhibited a unique pattern, with a significant increase after acute WS exposure compared to FA control (Fig. 7d). However, a notable decrease was observed when comparing to Sham mice in both FA and following acute WS exposure conditions. In contrast, the percentage of B cells in Sham-operated mice remained relatively consistent between FA and acute WS exposure conditions. These findings emphasize the distinctive role of OVX, combined with acute WS exposure (treatment), in shaping B cell responses, which aligns with an immunological clinical study that reported lower numbers of CD19 + B lymphocytes in a postmenopausal female patient cohort [70]. These results are also consistent with previous findings showing decreased CD19 + B lymphocytes and memory B cells (CD19 + CD27 +), elevated plasma IgE levels, and reduced production of CCL5 and TNF-α by monocyte-derived macrophages in biomass-smoke-exposed women [71, 72].

The findings also revealed significant alterations in T cells (CD3 + cells) and natural killer (NK) cells (NK1.1 + cells) in the bone marrow in OVX mice compared to Sham-operated mice, regardless of the exposure conditions (Fig. 7e,f, and Supplemental Fig S2f,g). This suggests that OVX has a substantial impact on bone marrow-derived immune cells. The cells in the bone marrow likely represent T-cell (lymphoid) progenitors, as only CD3 + cells were tested. While these are not necessarily memory T-cells and constitute a small percentage in the bone marrow, the data suggest they are not affected by WS-exposure. However, levels of CD3 + bone marrow-derived cells appear sensitive to ovarian hormone presence (as shown in Fig. 7e). Further research should elucidate the mechanisms of potential changes in T-cell populations with extended acute WS exposure and hormonal variations. T cells play a pivotal role in the mechanism of OVX-induced bone loss, as indicated in prior research, which showed increased circulating T cells and monocytes in postmenopausal women [73], as well as the lack of B cells in some models, and compensatory mechanisms such as an increase in NK cells producing the osteoclastogenic factor IL-17 [74], these findings shed light on the complex immune responses associated with OVX. Moreover, menopause, modeled in OVX mice, triggers the expression of activation markers in T cells, fostering T cell proliferation, expansion, and the acquisition of effector functions [75]. The mechanism underlying the expansion of TNF-producing T cells due to estrogen deficiency involves thymic function reactivation and T cell activation in the bone marrow, facilitated by enhanced antigen presentation by macrophages and dendritic cells (DCs) [76]. Atmospheric particles resulting from WS exposure induces the release of inflammatory cytokines, such as IL-1β, IL-6, and TNF-α, as well as chemokines and macrophages (regulated upon activation, normal T-cell expressed and secreted) and interferons [77, 78]. These cytokines facilitate the infiltration of neutrophils, dendritic cells (DCs), and T cells while also promoting the recruitment and maturation of monocytes from the bone marrow [79, 80]. Stromal cells demonstrated distinct changes following WS exposure in Sham mice, while monocytes/macrophages (Gr1low/CD11b + cells) exhibited notable responses in both Sham and OVX groups. Collectively, these findings underscore the intricate interplay of hormonal status, environmental exposures, and immune cell responses, providing a foundation for further research in understanding these complex interactions.

In summary, this study offers valuable insights into the complex relationship between acute wildfire smoke exposure, sex-based variations, and the influence of ovarian hormone status on lung health and immune responses. These findings hold significance for public health implications and future research within the realm of pulmonary and immunological medicine. Further investigations into the underlying mechanisms of this distinct response may illuminate the connection between hormonal fluctuations and immune reactions, thereby informing strategies to mitigate the health consequences during critical windows of susceptibility. Additionally, future studies might explore the potential impact of interventions like hormone replacement therapy (HRT) on modulating these responses in OVX mice, presenting possible avenues for immune system management in postmenopausal individuals facing exposure to environmental pollutants. All ovary-intact females in these studies were regularly cycling, as presented in Supplemental Table S1 [81]. For future investigations, evaluation of the estrous cycle on the day of each exposure or upon euthanasia, will enable the capture of nuanced variations across different estrous cycle phases [81], and aligning with prior research exploring analogous approaches and comparisons to post-menopausal stages.

## Conclusions

In summary, exposure to acute WS exposure has a multitude of adverse effects on systemic immunity [13]. Using a mouse model, this study highlights significant differences in proinflammatory cytokine and chemokine expression, as well as associated lung mRNA gene expression and the hematopoietic niche, between males and females. Additionally, distinctions were observed between females subjected to OVX and those who received sham surgery, both exposed to FA and acute wood smoke from wildfires. However, it is important to acknowledge the limitations of this study, specifically the utilization of whole lung tissue rather than specific cell types, which may be further explored by single-cell RNA-sequencing or spatial transcriptomics in the future, which could aid in identifying these specific cells influenced by these hormone and environmental factors. Another limitation of our study is the predisposition of C57BL/6 mice towards a skewed Th1-type cytokine response [82], which could potentially impact observed outcomes when compared to other strains. Given the contrasting Th2-type responses in BALB/c mice [82], future investigations could enhance scientific validity by incorporating diverse strains, such as BALB/c, to elucidate potential variations in responses to environmental pollutants. Moreover, given the 2-day exposure protocol consisting of daily 4-h periods, determining the peak of proinflammatory cytokine mRNA and protein is challenging without additional timepoint data. Further studies with more frequent sampling or additional timepoints are needed to accurately capture the temporal dynamics of cytokine expression following acute WS exposure. Overall, the findings from this research support the hypothesis of differential lung mRNA gene expression networks and potentially associated signaling pathways as mediators of sex differences and hormonal status in the inflammatory response of the lung to acute WS exposure during a wildfire event. Understanding the regulatory roles of differentially expressed genes in response to environmental insults holds significant implications, aiding in the identification of sex-specific therapeutic targets for acute lung inflammation and injury.

## Materials and methods

### Animals, ovariectomy surgery, and whole-body inhalation of acute Woodsmoke (WS)

Male and female C57BL/6 mice, as well as female Sham and OVX mice (*n* = 8 per treatment group) were purchased at 6–8 weeks of age from Taconic Biosciences (Albany, NY, USA). This age range was chosen for its suitability in biomedical research due to the well-characterized nature of the strain, reaching sexual maturity, and minimizing experimental variability [83]. Using C57BL/6 mice aged 6–8 weeks offers a robust and consistent model for investigating sex hormone effects on immune responses [83]. In a second set of experiments, female C57BL/6 mice (*n* = 8 per treatment group) were ovariectomized (OVX), which includes ovary removal, or received Sham surgery as controls. Mice were subjected to isoflurane anesthesia, and buprenorphine (0.01 mg/mL) was subcutaneously administered to both the right and left sides of the abdomen (0.2 mL/mouse in total). The incision site was sterilized with ethanol, followed by a betadine/iodine swab, after which the skin was delicately separated, connecting it with the back tissues using a blunt dissection technique. The ovaries were removed, fat pads replaced, and internal structures positioned in the original positions with a single stitch used to close the peritoneal incision. Mice were then returned to the recovery cage and vigilantly observed for 10 continuous days for indications of distress and their incision recovery progress. For the Sham control group, mice received Sham surgery performed identically to the OVX surgery, except that the ovaries were left intact, and the incision was subsequently sutured. Sham mice were then allowed to recover in a manner similar to that of the OVX mice.

After a post-operative recovery phase and a 2-week acclimation period in suitable housing facilities, in accordance with the university-approved institutional animal care and use committee (IACUC) protocol, all mice, which included both male and female C57BL/6 mice, along with female C57BL/6 mice that had undergone Sham or OVX procedures, were transferred to inhalation exposure chambers. They were then assigned to a 2-day exposure regimen that included daily 4-h intervals of acute WS at a concentration of approximately 0.575 ± 0.12 mg/m^3^, [83] which was a setup that emulated an acute exposure to acute WS from a wildfire event, and consistent with real-world exposure levels, which were measured to be 0.5 mg/m^3^, [83] or to HEPA-filtered air (FA) in a HEPA-FA chamber for the same timeframe as WS exposures, which served as the control condition (*n* = *8* per group; Fig. 1). Inhaled WS exposure were facilitated by vacuum and pressurization and total pressure monitoring did not exceed ± 25 mmHg within the chamber. Concentrations were periodically monitored and manually adjusted by the user to ensure consistent exposure concentrations. Mice across all groups were euthanized, and tissues were collected approximately 24 h following the completion of the exposure. Woodsmoke exposure comprised a 2-g burned biomass wood sample, sourced from local piñon trees in the Southwestern United States.

### Metal analysis of particulate matter in filter samples using Inductively Coupled Plasma Mass Spectrometry (ICP-MS)

Gravimetric samples were collected on Teflon filters after inhalation exposure, and these filters were then subjected to trace metals analysis by ICP-MS in triplicate for each sample. Each filter was immersed in 3 mL, 70% trace metal-grade nitric acid (Aristar Plus grade, BDH), and the samples were heated for 2 h at 373.15 K to fully digest the particulates. In one batch, the filters were squeezed as dry as possible and then removed. The remaining solutions were then diluted using 18 M-ohm water prior to ICP-MS analysis. In the other batch, the samples were diluted with 18 M-ohm water without removing the filters and fully vortexed, and the supernatants were extracted for ICP-MS analysis. The Agilent 7900 ICP-MS instrument was optimized using a multi-element tuning solution spanning a wide mass range. Calibration curves were established for each element of interest based on blank samples and seven standards, with all measurements performed in triplicate and under Helium [He] Mode to reduce potential interferences. Quality control samples were systematically integrated into the data acquisition and data analysis process to ensure data accuracy and reliability.

### Real-time Quantitative Polymerase Chain Reaction (RT-qPCR) gene expression

Real-time quantitative polymerase chain reaction (RT-qPCR) was used to assess normalized gene expression in the lungs. Briefly, lungs were dissected following euthanasia of the animals and snap-frozen in liquid nitrogen. Following the liquid nitrogen, samples were transferred to − 80 ^◦^C storage until further use. A commercial RNA kit (RNeasy, Qiagen, Germantown, MD, USA) was used to extract total RNA from each sample. Samples were reverse-transcribed with High-Capacity cDNA Reverse Transcription reagents (Applied Biosystems, Foster City, CA, USA). Gene expression was then assessed using iTaq Universal SYBR Green Supermix (Biorad, Hercules, CA, USA) and a 385 CFX Opus Real-Time PCR machine (Bio-Rad, Hercules, CA, USA). Mouse primers (Qiagen, Redwood City, CA, USA) included, *IL-1b* (QT01048355), *CCL-2* (QT00167832, *TNF-a* (QT00116564), *CXCL-1* (QT00115647), *CCL-5* (QT01747165), *TGF-b* (QT00145250), *IL-6* (QT00098875) and 18 s rRNA (QT02448075), as the housekeeping gene. Relative gene expression using these target genes was analyzed using the 2^−∆∆CT^ method, and FA groups were normalized to 1 [84].

### Collection of bronchoalveolar lavage cells, plasma, and bone marrow following whole-body inhalation exposure

After exposure, mice were returned to their cages overnight at the Animal Research Facility at the University of New Mexico. For plasma collection, mice were deeply sedated with isoflurane, and whole blood was directly collected from the heart via cardiac puncture using a 23-gauge needle. The collected blood samples were placed into EDTA plasma separator tubes to prevent clotting by chelating calcium ions. Subsequently, the blood samples were left for 15–20 min at 293–298 K (room temperature) before being transferred to an ice-cooled environment to preserve their integrity, followed by centrifugation for 10 min at 1200* g* to separate the plasma from the distinct blood components. The plasma from the top layer was collected into a new pre-labeled cryovial and kept frozen at 193.15 K until it was prepared for further cytokine characterization.

Following exsanguination, BALF was collected from the mouse lungs. Briefly, a cannula was aseptically inserted into the trachea of each mouse under continuous isoflurane anesthesia, according to approved IACUC protocols. BALF was collected by flushing the lungs with 1 mL of phosphate-buffered saline (PBS, Thermo Fisher Scientific, WA, USA) and aspirating the fluid using a 1 mL syringe fitted onto the cannula, with the volume of returned BALF recorded. The collected BALF was then transferred into an Eppendorf tube, kept on ice, and counted after a 1:5 dilution (15 µL saline + 10 µL BALF + 25 µL trypan blue stain) using an automated cell counter. Cytospins were prepared using a cell calculator, wherein 250–300 µL of BALF was centrifuged for 5 min at 800 rpm to be applied directly onto cytospin slides. The slides were then air-dried at 293–298 K (room temperature) until differential (dff-quick) staining. The remaining BALF was pelleted by centrifugation for 2 min at 14,000* g* and 293–298 K, with the supernatant collected into new pre-labeled cryovials, all of which was then stored at 193.15 K until further cytokine analysis. Total cells, which include BALF polymorphonuclear neutrophils (PMNs) and macrophages, were stained using Hoechst stain (Thermo Fisher Scientific, WA, USA), quantified, and presented as a percentage of total cells.

Total bone marrow was flushed from femurs and tibiae of mice, as previously described [85]. Intact femurs and tibias were harvested and immediately placed in Eppendorf tubes on ice for temporary storage. The bones were meticulously cleaned of tissue debris using tweezers, scissors, and gauze, after which a small tip of bone on both sides was clipped off. The cleaned bones were then inserted vertically into 0.5 mL microcentrifuge Eppendorf tubes, each of which had a hole in the bottom created using an 18-G, 1.5-in. needle. These 0.5 mL tubes were nested within 1.5 mL tubes and centrifuged for 15 s at ≥ 10,000* g*. The successful separation of bone marrow from the bones was confirmed through visual inspection, where the bones exhibited a white appearance, and a distinct pellet of bone marrow spun out. The 0.5 mL tube containing the bones was then discarded, and the bone marrow pellet was re-suspended in 1 mL of freeze media (95% FBS + 5% DMSO). Following this step, 0.5 mL aliquots were prepared in two separate pre-labeled cryovials, with an additional 0.5 mL of freeze media added to each cryovial to reach a total volume of 1 mL. These cryovials were stored in a 193.15 K freezer until further analysis by flow cytometry.

### Bronchoalveolar lavage and plasma cytokine detection

To evaluate cytokine expression in collected BALF and plasma samples following FA control and acute WS exposures, the V-Plex Proinflammatory Panel 1 mouse kit (K15048D-1, Meso Scale Diagnostics, Rockville, MD, USA) was used. Cytokines, which included IFN-γ, IL-10, IL-6, IL-2, KC-GRO, IL-1β, IL-5, and TNF-α, were assessed and expressed as pg/mL, according to the manufacturer's instructions for Meso Scale kits, with appropriate sample dilutions for plasma. BALF samples were not diluted and detected cytokine levels fell within the required standard curve. Briefly, plates were incubated with the samples and gently shaken for 2 h at 298 K, followed by three successive washes with a buffered solution. Detection antibodies were subsequently added to the wells and incubated for 1 h at 298 K. Read buffer was introduced to the wells, and plates were analyzed using a Meso Scale Discovery QuickPlex SQ instrument (Meso Scale Diagnostics, Rockville, MD, USA). Discovery Workbench software was then used to calculate pg/mL cytokine concentrations in each biofluid, based on the standard curve for each cytokine.

### Flow cytometry analysis of bone marrow

A total of ${1\text{ x }10}^{6}$ bone marrow cells was stained for cell surface makers using two flow cytometry panels. The bone marrow cells were initially blocked with rat anti-mouse CD16/CD32 Fc receptor block (BD Biosciences 553,141, San Jose, CA), followed by staining with fluorochrome-conjugated primary antibodies specific for cell surface markers listed in Table 1. This staining process was carried out in fluorescence-activated cell sorting (FACS) buffer (1 × PBS) supplemented with 5% fetal bovine serum (FBS; R&D Systems, Minneapolis, MN) and 10 mM sodium azide. The assessed cell types included the percentage of total white blood cells (CD45 +), neutrophils (Gr1 +), myeloid cells, including dendritic cells (CD11c +), B-cells (CD19 +), T-cells (CD3 +), NK cells (NK1.1 +), monocytes/macrophages (Gr1low/CD11b +), and stromal cells (CD45-/CD44 +), with the gating schematic shown in Supplemental Fig S2. The percentages of immune cell populations were reported as a fraction of CD45 + cells. Data analysis was conducted using an NxT Attune V6 Flow Cytometer (ThermoFisher, Waltham, MA) and FlowJo Software (BD Biosciences).

**Table 1:** Fluorochrome-conjugated primary antibodies for flow cytometry analysis

Fluorochrome	Antibody	Concentration (μg/RXN)	Company	Cat #
BV605	CD45	0.4	BD Biosciences	563,053
V450 or eF450	CD11b	0.0025	BD Biosciences	560,455
FITC	Gr1	0.25	BD Biosciences	553,127
PerCP-Cy5.5	CD11c	0.0375	BD Biosciences	560,584
AF488	CD44	0.25	BioLegend	103,016
PE	Nk1.1	0.2	BD Biosciences	557,391
PerCP-Cy5.5	CD3e	0.2	BD Biosciences	551,163
APC	CD19	0.4	BD Biosciences	550,992

### Statistical analysis

This study was designed as a 2 × 2 factorial two-way analysis of variance (ANOVA) with sex (C57BL/6 males vs. females) or surgery (Sham-operated vs. OVX females) as the first factor, and treatment (control group with FA vs. group exposed to acute WS) as the second factor. Statistical significance was determined to assess the impact of the following factors: sex, surgery, treatment, and interactions between sex and treatment or between surgery and treatment, on lung mRNA gene expressions, BALF cytokine levels, and immune cell responses. These interactions were examined using a two-way ANOVA, followed by Tukey's post-hoc tests, which identified statistically significant mean differences between groups. The results were presented as means ± standard error of the mean (SEM), with statistical significance set at *P* < *0.05*. Data were graphed using GraphPad Prism software (GraphPad Holdings, LLC, San Diego, CA, USA).

### Supplementary Information

Supplementary Material 1: Supplemental Figure S1. Real-time monitoring of concentrations (ppm, mg/m^3^) of nitric oxide (NO), carbon monoxide (CO), and ozone (O_3_) gases in the woodsmoke (WS) exposure chamber. Woodsmoke exposure system operated overnight at a chamber temperature range of 21.3 – 21.5 °C, with samples collected every 10 minutes using the Grey Wolf TG501 gas sensor.

Supplementary Material 2: Supplemental Figure S2. Flow cytometry gating strategy for assessing the ovariectomy-dependent response of bone marrow-derived cells to acute WS exposure. Scatter plots represent the impact of acute WS exposure on various bone marrow-derived cellular subpopulations in OVX and Sham mice, as shown in Fig 7, which include the percentages of (a) total white blood cells (CD45+ cells), (b) neutrophils (Gr1+ cells), (c) myeloid cells, including dendritic cells (CD11c+ cells), (d) stromal cells (CD45-/CD44+ cells), (e) B-cells (CD19+ cells), (f) T-cells (CD3+ cells), (g) NK cells (NK1.1+ cells), and (h) monocytes/macrophages (Gr1low/CD11b+ cells). These subpopulations are discerned through the use of primary antibodies that are fluorochrome-conjugated and specific to the cell surface markers outlined in Table 1. Each treatment group had a total of *n*=8 mice. Mice across all groups were euthanized 24 hours post-exposures.

Supplementary Material 3: Supplemental Table S1. Estrous cycle data immediately prior to exposures in female mice. Vaginal cytology was used to assess estrous cycle data for several days prior to exposure. All female mice exposed to FA or WS were regularly cycling. Proestrus (P), estrus (E), metestrus (M), and diestrus (D) was monitored each day prior to exposure.

## Data Availability

No datasets were generated or analysed during the current study.
