# Supplementary figures and images for: Systemic immunological responses are dependent on sex and ovarian hormone presence following acute inhaled woodsmoke exposure
Source: Part Fibre Toxicol. 2024 May 27;21:27. doi: 10.1186/s12989-024-00587-5 (PMC11129474; doi:10.1186/s12989-024-00587-5)

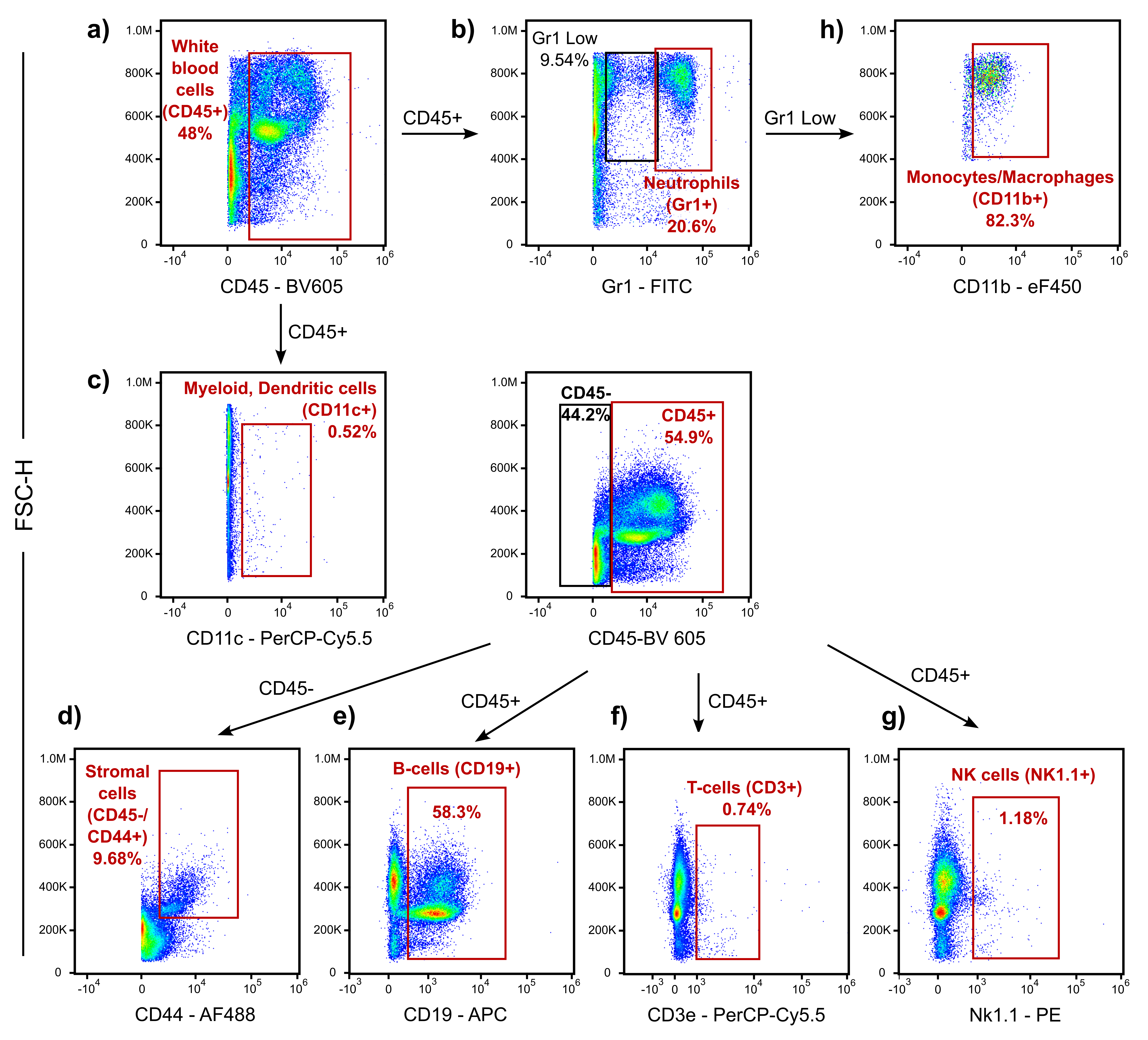

Supplement: Supplementary file 2 — Supplementary Material 2: Supplemental Figure S2. Flow cytometry gating strategy for assessing the ovariectomy-dependent response of bone marrow-derived cells to acute WS exposure. Scatter plots represent the impact of acute WS exposure on various bone marrow-derived cellular subpopulations in OVX and Sham mice, as shown in Fig 7, which include the percentages of (a) total white blood cells (CD45+ cells), (b) neutrophils (Gr1+ cells), (c) myeloid cells, including dendritic cells (CD11c+ cells), (d) stromal cells (CD45-/CD44+ cells), (e) B-cells (CD19+ cells), (f) T-cells (CD3+ cells), (g) NK cells (NK1.1+ cells), and (h) monocytes/macrophages (Gr1low/CD11b+ cells). These subpopulations are discerned through the use of primary antibodies that are fluorochrome-conjugated and specific to the cell surface markers outlined in Table 1. Each treatment group had a total of n=8 mice. Mice across all groups were euthanized 24 hours post-exposures. [file 12989_2024_587_MOESM2_ESM.png]
